# Biologically effective dose (BED) of stereotactic body radiation therapy (SBRT) was an important factor of therapeutic efficacy in patients with hepatocellular carcinoma (≤5 cm)

**DOI:** 10.1186/s12885-019-6063-9

**Published:** 2019-08-28

**Authors:** Jing Sun, Tao Zhang, Jia Wang, Wengang Li, Aimin Zhang, Weiping He, Dan Zhang, Dong Li, Junqiang Ding, Xuezhang Duan

**Affiliations:** Radiation Oncology Center, The Fifth Medical Center of PLA General Hospital (Beijing 302 Hospital), No. 100 Xi Si Huan Middle Road, Fengtai District, Beijing, 100039 China

**Keywords:** Hepatocellular carcinoma, Stereotactic body radiation therapy, Radiation-induced injury, Biologically effective dose, Platelet

## Abstract

**Background:**

To explore the association between biologically effective dose (BED) and survival rates in Child-Pugh A classification (CP-A) small hepatocellular carcinoma (HCC) patients treated with stereotactic body radiation therapy (SBRT).

**Methods:**

This retrospective study included 108 small HCC patients who were treated with SBRT between 2011 and 2014. The prescribed dose delivered to the tumor were 48Gy/8f, 49Gy/7f, 50Gy/5f and 54Gy/6f. The median biologically effective dose (BED_10_) of the total prescribed dose was 100Gy (76.8–102.6Gy). Factors associated with the survival rate were examined using the Cox proportion hazards model, and the factors associated with radiation-induced liver injury (RILD) were examined by logistic regression analysis.

**Results:**

For these patients, the median follow-up time was 42 months (6–77 months), and the 1-, 2- and 3-year overall survival (OS) rates were 96.3, 89.8 and 80.6%, respectively. The 1-, 2- and 3-year progression-free survival (PFS) rates were 85.2, 70.1 and 60.6%, respectively. The 1-, 2- and 3-year local control (LC) rates were 98.1, 96.2 and 95.1%, respectively. The 1-, 2- and 3-year distant metastasis- free survival (DMFS) rates were 86.1, 72.8 and 61.2%. The OS, PFS and DMFS were significantly higher in the BED_10_ ≥ 100Gy group than in the BED_10_ < 100Gy group (OS: *p* = 0.020; PFS: *p* = 0.017; DMFS: *p* = 0.012). The PLT count was a predictive factor of RILD.

**Conclusions:**

SBRT is a safe and effective option for CP-A HCC patients. A BED_10_ value greater than 100Gy and lower CP score are associated with improved OS and PFS. Additionally, the peripheral PLT count are predictive factors of RILD.

**Electronic supplementary material:**

The online version of this article (10.1186/s12885-019-6063-9) contains supplementary material, which is available to authorized users.

## Background

Hepatocellular carcinoma (HCC) is the sixth most common malignancy and the third most common cause of cancer mortality worldwide, and only approximately 20–30% of patients with HCC are eligible for surgical treatment, including liver resection and liver transplantation [1, 2]. Accumulating data have shown that stereotactic body radiation therapy (SBRT) is a safe and effective treatment for HCC, especially in patients with inoperable or recurrent HCC [3–5]. Furthermore, Su et al. [6] compared the efficacy of stereotactic ablative radiation therapy (SABR) versus liver resection for treating small HCC (< 5 cm) patients with Child-Pugh class A (CP-A) cirrhosis and concluded that SABR has local effects that are similar to those of liver resection. Wahl et al. [7] reported that SBRT and radiofrequency ablation (RFA) were equally effective for treating small HCCs. We conducted this retrospective study to evaluate the efficacy of SBRT and identify prognostic factors related to the efficacy of SBRT in patients with HCC.

In other cancers, such as lung and cervical cancers, with the delivery of increasing biologically effective doses (BEDs) to lesions, the OS of patients increased [8–10]. However, due to the scarcity of data on HCC, the relationship between the BEDs and the efficacy of SBRT in HCC patients was included in our study.

Radiation-induced liver injury (RILD) is often a fatal complication of SBRT and should be avoided in patients with HCC; furthermore, the CP classification is an important predictive factor of RILD [11–13]. Therefore, we investigated the incidence of RILD in the included patients and searched for predictive factors among their clinical data, including biochemical parameters and the peripheral platelet (PLT) and white blood cell (WBC) counts.

## Methods

### Enrolled patients’ characteristics and SBRT parameters

We conducted a retrospective observation of CP-A HCC patients. The eligibility criteria were the following: (a) primary HCC diagnosed by a surgeon and/or radiologist and oncologist according to the international guidelines for the management of HCC or by pathology [14]; (b) single lesion and longest tumor diameter < 5.0 cm; (c) CP-A classification; (d) Eastern Cooperative Oncology Group (ECOG) score 0–1; (e) distances between tumor and normal organs (esophagus, stomach, duodenum, bowel) were more than 5 mm; (f) unsuitable for other therapies, such as patients with heart disease, uncontrolled diabetes, uncontrolled hypertension, etc. (g) rejecting other therapies such as resection, liver transplantation, etc. (h) platelet count≥50 × 10^9^/L, white blood count≥1.5 × 10^9^/L and (i) patients infected with hepatitis B virus who were treated with adefovir or entecavir; patients infected with hepatitis C virus whose HCV DNA were negative. The exclusion criteria were the following: (a) tumor thrombus; (b) lymph node involvement; and (c) extrahepatic metastasis. Patients were treated with SBRT at The Fifth Medical Center of PLA General Hospital between 2011 and 2014. They did not receive any other treatments, such as RFA or transcatheter arterial chemoembolization (TACE) before enrollment into our study. All patients were managed in multidisciplinary setting with all legitimate treatment options available and provided with written informed consent before treatment.

The baseline data of the 108 patients in our study are listed in Table 1.ALBI parameter in this study was calculated from baseline blood work according to Johnson et al. [15]. ALBI = [log_10_bilirubin × 0.66] + [albumin× (− 0.085)], where bilirubin is in μmol/L and albumin is in g/L; the cutoffs were used to assign each patient to one of three prognostic groups indicating the ALBI grade (range, 1 to 3). The cutoffs were as follows: xb ≤ − 2.60 (ALBI grade 1), > − 2.60 to ≤1.39 (ALBI grade 2), and > − 1.39 (ALBI grade 3).

**Table 1:** Clinical, biochemical characteristics and radiation planning parameters of patients enrolled in this study

Variables	n
Clinical and biochemical characteristics	
Sex	
Male	80 (74.07%)
Female	28 (25.93%)
Age (years)	
Median	54
Range	37–77
Underlying liver disease	
Hepatitis B	97 (89.81%)
Hepatitis C	7 (6.48%)
Alcoholic hepatitis	3 (2.78%)
None	1 (0.93%)
Maximum tumor diameter (cm)	
Median	2.3
Range	0.7–4.9
AFP (ng/ml) ^*a*^	
Median	33.13
Range	0.8–7896
Child-Pugh score	
5	97 (89.81%)
6	11 (10.19%)
ALBI grade	
Grade 1	34 (31.48%)
Grade 2	74 (68.52%)
WBC count (×10^9^/L)	
Median	4.86
Range	1.53–9.4
PLT count (×10^9^/L)	
Median	118
Range	50–283
Radiation planning parameters	
Isodose line of maximum dose	
Median (range, %)	72 (60–86)
Residual normal liver volume	
Median (range, cm^3^)	1324 (834–2493)
Mean dose of whole liver volume minus GTV	
Median (range, Gy)	9.07 (4.04–12.05)
Dose received by volume of liver	
D700^c^	4.99 (1.40–13.50)
BED_10_^b^ of Plan target volume (PTV)	
Median (range, Gy)	100 (76.8–102.6)

### Radiation treatment technique

All enrolled patients underwent the implantation of 4 to 6 fiducials one week prior to SBRT (CyberKnife, Accuray, USA). All plans were designed using G4 CyberKnife MultiPlan (version 4.0.2) and were applied with dynamic respiration tracking combined with fiducial tracking.

An oncologist contoured the gross tumor volume (GTV) and organs at risk (normal liver, kidneys, esophagus, stomach, duodenum, bowel and spinal cord). The planning target volume (PTV) was expanded 3–5 mm around the GTV, which contoured 100% of GTV. The prescribed dose delivered to the tumor were 48Gy/8f, 49Gy/7f, 50Gy/5f and 54Gy/6f. The selection of dose depended on the relation between lesion and bile duct. If the distance between tumor and bile duct was less than 3 mm, the prescribed dose delivered to the tumor were 48Gy/8f or 49Gy/7f. The normal tissue dose was within the normal radiotherapy tolerance dose (TG-101) [16]. In this study, both the single dose and the total dose varied, so the BED was chosen as the parameter of reaction dose fractionated schemes.

The BED was calculated according to the value of α/β (10Gy, BED_10_) using the formula BED = D (1 + d/[α/β]) [17, 18], where D is the total dose delivered, and d is the dose per fraction. The median BED_10_ of the total prescribed dose was 100Gy (76.8–102.6Gy) in our research.

### Follow-up study

All patients underwent a liver function assessment and routine blood examinations before treatment. After SBRT, the patients were followed every 3 months for 1 year and every 6 months thereafter until June 2018.

### Toxicity evaluation

Radiation-related toxicity was measured based on Toxicity criteria of the Radiation Therapy Oncology Group (RTOG) and the European organization for research and treatment of cancer (EORTC) [19].

The liver toxicity reaction evaluation was based on the definition of RILD, of which there are two types: classic RILD and non-classic RILD.

Classic RILD usually manifests as symptoms of fatigue, hepatomegaly, and anicteric ascites, etc., 1–3 months after radiotherapy. Moreover, the serum alkaline phosphatase (ALP) level in these patients increases to more than twice the normal level, while the serum transaminase and bilirubin levels in remain normal [20, 21].

Non-classic RILD occurs in patients with underlying chronic hepatic diseases, who suffer from jaundice and/or remarkably elevated serum transaminase levels (increased by more than fivefold compared to normal levels) [22, 23].

Meanwhile, CP score progression (increasing by 2 or more scores) was also a clinical metric for non-classic RILD [24] .

### Tumor recurrence and treatment

When the recurrence/metastasis was confirmed, second-line treatments were individualized according to the number and location of the recurrent tumors and the liver function status, while considering the patient’s preferences. Therapeutic options included repeated SBRT, targeted therapy and conservative treatment.

### Statistical analysis

OS was calculated starting from the date of SBRT to the date of the final follow-up or demise of the patients. PFS was estimated starting from the date of SBRT to the date of disease progression or patient death. LC was defined starting from the date of SBRT to the date of treated-lesion progression or patient death. DMFS was defined starting from the date of SBRT to the date of distant metastasis occurrence (out-field relapse). OS, PFS, LC and DMFS were estimated using the Kaplan-Meier method. OS-, PFS-, LC- and DMFS- related group analyses of BED_10_ were performed using the log rank test. Univariate and multivariable hazard ratios were calculated using the Cox proportion hazard model. A binary logistic regression method was employed to investigate each prognostic factor of RILD. Variables with *p*-values less than 0.2 in the univariate analysis were included in the multivariate analysis with forward selection. For comparisons between the baseline variables, the χ2 test and Fisher’s exact test were performed.

All statistical analyses were performed using SPSS 22.0 software (IBM) and STATA 15.0 software. *P* values < 0.05 were considered statistically significant.

## Results

**Fig. 1:**
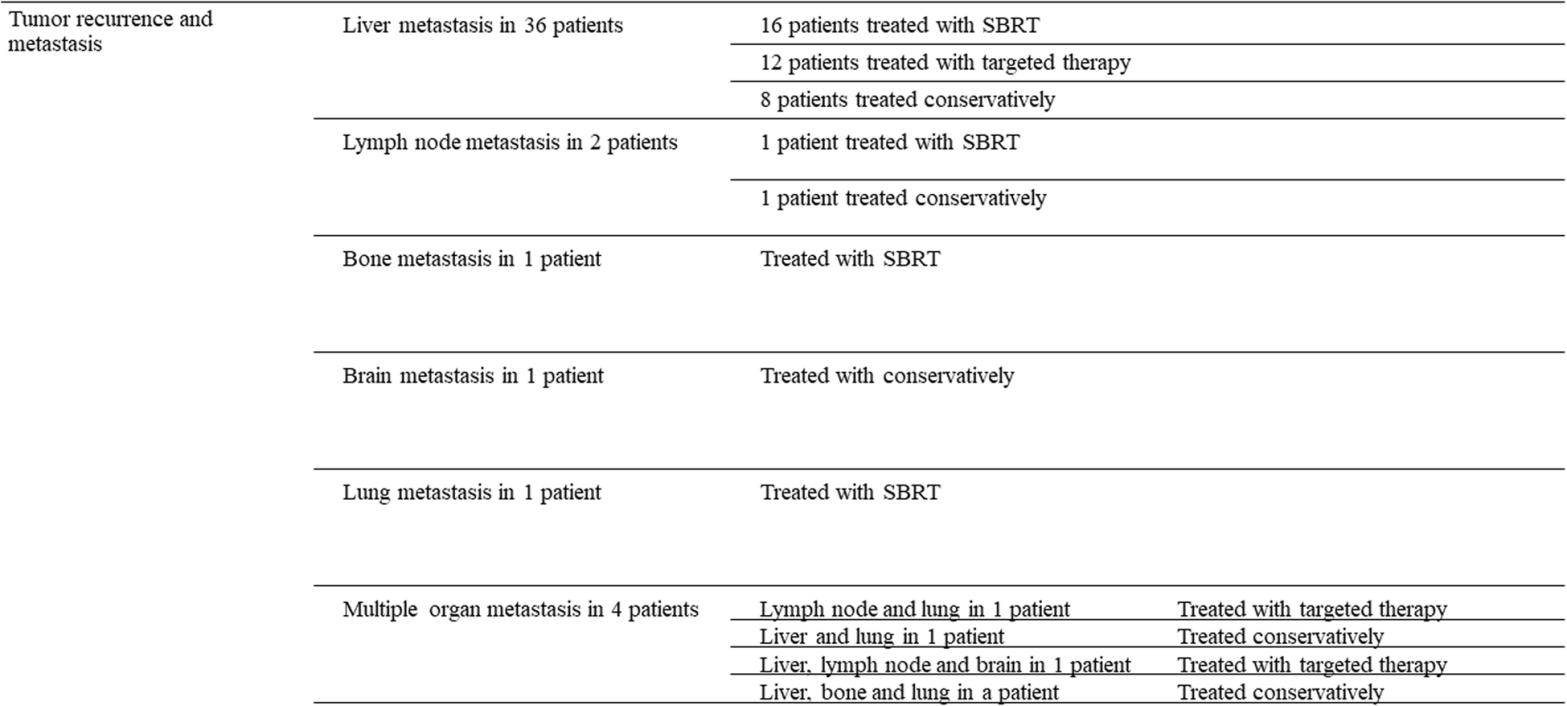
The treatment was selected by the patient

The median follow-up period was 42 months (range, 6–77 months). By June 2018, 45 patients had experienced relapse or metastasis; 36 patients experienced liver metastasis, 2 experienced lymph node metastasis, 1 experienced bone metastasis, 1 experienced brain metastasis, 1 experienced lung metastasis and 4 experienced multiple organ metastases. The treatment was ultimately selected by the patient (Fig. 1).

### Survival outcomes

After 6 months of SBRT, there were 65 patients with CR (60.19%), 30 patients with PR (27.78%), 4 patients with SD (3.70%) and 9 patients with PD (8.33%). The response rate was (CR + PR)/108 × 100% = 87.96%, and the disease control rate was (CR + PR + SD)/ 108 × 100% = 91.67%.

By June 2018, 27 patients died: 6 patients died of hepatic failure; 6 died of upper gastrointestinal bleeding; 1 died of hepatorenal syndrome; 1 died of infectious shock; 1 died of pulmonary or brain metastasis complications; and 12 died of unknown causes.

**Fig. 2:**
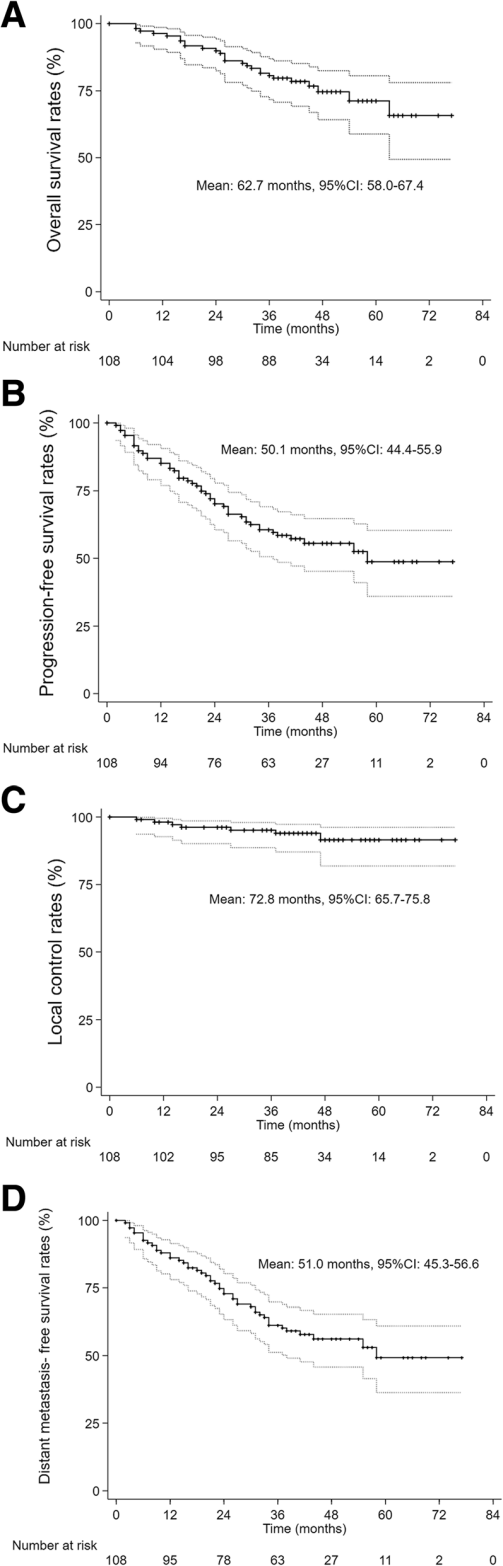
Kaplan-Meier curve of overall survival (**a**), progression-free survival (**b**), local control (**c**) and distant metastasis-free survival (**d**)

The 1-, 2- and 3-year OS rates were 96.3, 89.8 and 80.6%, respectively (Fig. 2a). The 1-, 2- and 3-year PFS rates were 85.2, 70.1 and 60.6%, respectively (Fig. 2b). The 1-, 2- and 3-year LC rates were 98.1, 96.2 and 95.1%, respectively (Fig. 2c).

The 1-, 2- and 3-year DMFS rates were 86.1, 72.8 and 61.2%, respectively (Fig. 2d). The 5-year cumulative OS, PFS, LC and DMFS were 71.2,48.7,91.5 and 49.2%, respectively.

In the Cox proportional hazard model, the Child-Pugh score and BED_10_ were independent prognostic factors of OS (Table 2), PFS (Table 3) and DMFS (Table 4) on multivariate analysis.

**Table 2:** Univariate and multivariate Cox regression analysis of OS

	Univariate Cox regression		Multivariate Cox regression	
Patient details	*p* value	Hazard ratio (95% CI)	*p* value	Hazard ratio (95% CI)
Sex (male/female)	0.214	1.856 (0.700–4.922)		
Age	0.983	1.000 (0.960–1.043)		
Hepatitis type (B/C/alcoholic/others)	0.645	1.167 (0.606–2.247)		
Maximum tumor diameter	0.368	1.222 (0.790–1.889)		
ECOG PS	0.432	1.621 (0.486–5.402)		
AFP	0.968	1.000 (1.000–1.000)		
Child-Pugh score (5/6)	0.095	2.300 (0.866–6.108)	0.048	2.740 (1.011–7.425)
ALBI grade	0.708	1.156 (0.542–2.465)		
WBC count	0.181	0.822 (0.618–1.095)		
PLT count	0.134	0.993 (0.984–1.002)		
Residual normal liver volume	0.375	0.999 (0.997–1.001)		
Mean dose of whole liver volume minus GTV	0.810	0.997 (0.972–1.022)		
D700	0.252	1.151 (0.905–1.463)		
BED_10_ of PTV	0.030	0.960 (0.925–0.996)	0.017	0.955 (0.919–0.992)

**Table 3:** Univariate and multivariate Cox regression analysis of PFS

	Univariate Cox regression		Multivariate Cox regression	
Patient details	*p* value	Hazard ratio (95% CI)	*p* value	Hazard ratio (95% CI)
Sex (male/female)	0.094	1.864 (0.900–3.858)		
Age	0.673	0.993 (0.962–1.025)		
Hepatitis type (B/C/alcoholic/others)	0.043	1.546 (1.013–2.359)		
Maximum tumor diameter	0.848	1.026 (0.789–1.334)		
ECOG PS	0.388	1.505 (0.595–3.805)		
AFP	0.440	1.000 (1.000–1.000)		
Child-Pugh (5/6)	0.071	2.100 (0.938–4.071)	0.029	2.500 (1.096–5.705)
ALBI grade	0.156	0.656 (0.367–1.174)		
WBC count	0.051	0.850 (0.723–1.001)		
PLT count	0.997	0.993 (0.992–1.003)		
Residual normal liver volume	0.375	0.999 (0.997–1.001)		
Mean dose of whole liver volume minus GTV	0.524	0.959 (0.845–1.090)		
D700	0.839	1.018 (0.857–1.210)		
BED_10_ of PTV	0.020	2.063 (1.120–3.803)	0.020	2.063 (1.120–3.803)

**Table 4:** Univariate and multivariate Cox regression analysis of DMFS

	Univariate Cox regression		Multivariate Cox regression	
Patient details	*p* value	Hazard ratio (95% CI)	*p* value	Hazard ratio (95% CI)
Sex (male/female)	0.114	1.801 (0.868–3.736)		
Age	0.777	0.995 (0.964–1.028)		
Hepatitis type (B/C/alcoholic/others)	0.080	1.668 (0.940–1.028)		
Maximum tumor diameter	0.901	1.017 (0.780–1.326)		
ECOG	0.220	1.799 (0.704–4.594)		
AFP	0.471	1.000 (1.000–1.000)		
Child-Pugh score (5/6)	0.054	2.214 (0.996–4.965)	0.021	2.653 (1.160–6.067)
ALBI grade	0.487	0.714 (0.277–1.843)		
WBC count	0.061	0.854 (0.724–1.007)		
PLT count	0.320	0.997 (0.992–1.003)		
Residual normal liver volume	0.982	1.000 (0.998–1.002)		
Mean dose of whole liver volume minus GTV	0.898	0.993 (0.886–1.112)		
D700	0.496	1.064 (0.889–1.274)		
BED_10_ of PTV	0.013	0.964 (0.937–0.992)	0.006	0.959 (0.932–0.988)

### Comparison between BED_10_ ≥ 100Gy and BED_10_ < 100Gy

**Fig. 3:**
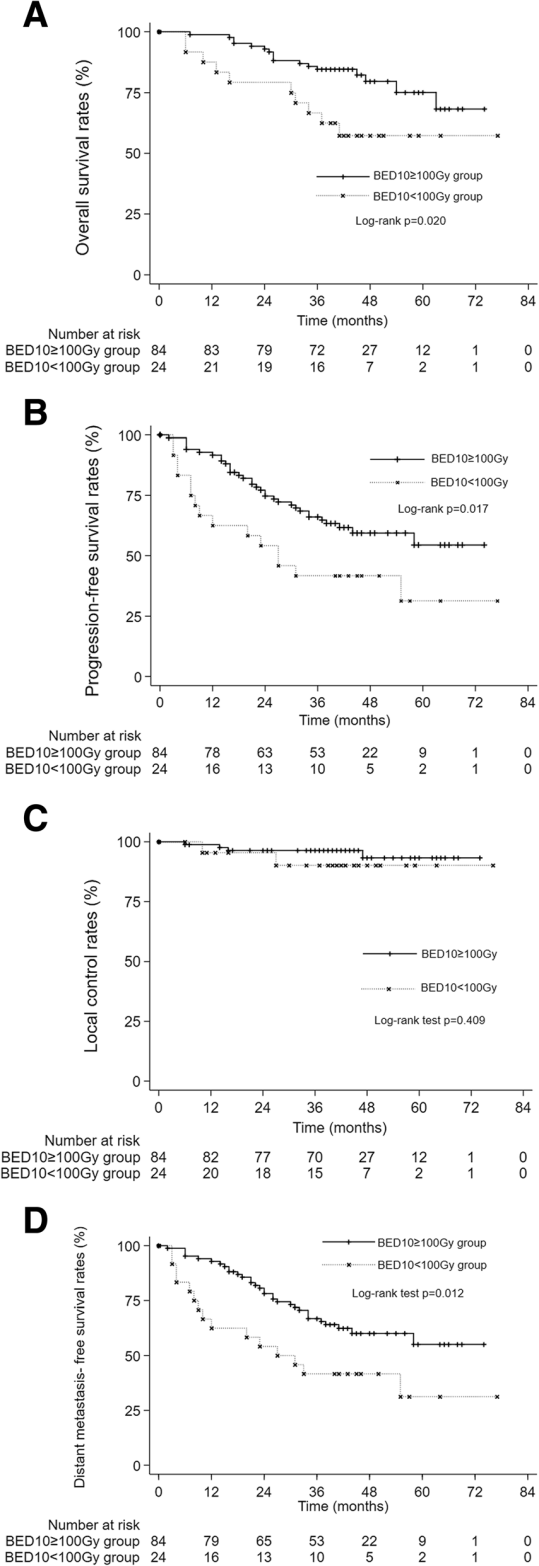
A OS in the BED_10_ ≥ 100 Gy and BED_10_ < 100 Gy groups. The 1-, 2- and 3-year OS rates were 98.8, 92.9 and 84.5% in the BED_10_ ≥ 100 Gy group and 87.5, 79.2 and 66.7% in the BED_10_ < 100 Gy group, respectively (*p* = 0.020). B PFS in the BED_10_ ≥ 100 Gy and BED_10_ < 100 Gy groups. The 1-, 2- and 3-year PFS rates were 91.7, 74.7 and 66.0% in the BED_10_ ≥ 100 Gy group and 62.5, 54.2 and 41.7% in the BED_10_ < 100 Gy group, respectively (*p* = 0. 017). C LC in the BED_10_ ≥ 100 Gy and BED_10_ < 100 Gy groups. The 1-, 2- and 3-year LC rates were 98.8, 96.4 and 96.4% in the BED_10_ ≥ 100 Gy group and 95.5, 95.5 and 90.2% in the BED_10_ < 100 Gy group, respectively (*p* = 0.409). D DMFS in the BED_10_ ≥ 100 Gy and BED_10_ < 100 Gy groups. The 1-, 2- and 3-year DMFS rates were 92.9, 80.7 and 66.9% in the BED_10_ ≥ 100 Gy group and 62.5, 54.2 and 41.7% in the BED_10_ < 100 Gy group, respectively (*p* = 0.012)

To further examine the BED_10_, we divided the patients into two groups with a BED_10_ of 100Gy as the cutoff, i.e., the BED_10_ ≥ 100Gy and BED_10_ < 100Gy groups. There were no differences in the detail of the patients between the two groups (Table 5). There were 84 patients in the BED_10_ ≥ 100Gy group and 24 patients in the BED_10_ < 100Gy group. The OS, PFS and DMFS rates were significantly higher in the BED_10_ ≥ 100Gy group than in the BED_10_ < 100Gy group (OS: *p* = 0.020, Fig. 3a; PFS: *p* = 0.017, Fig. 3b; DMFS: *p* = 0.012, Fig. 3d). However, there were no significant differences in LC between the two groups (*p* = 0.409, Fig. 3c).

**Table 5:** Details of the patients in the two groups

	BED_10_ ≥ 100 Gy	BED_10_ < 100 Gy	
Details			*p* value
Sex (male/female)	62/22	18/6	0.907
Age	54.33 ± 8.82	54.60 ± 11.59	0.885
Maximum tumor diameter	2.50 ± 1.02	2.74 ± 1.10	0.406
ECOG PS (0/1)	78/6	22/2	0.844
Child-Pugh score (5/6)	75/9	22/2	0.734
ALBI grade (1/2)	25/59	9/15	0.472
WBC count	5.00 ± 1.99	4.68 ± 1.51	0.528
PLT count	127.06 ± 59.06	112.04 ± 39.47	0.244
Residual normal liver volume	1345.33 ± 286.81	1390.36 ± 329.11	0.614
D700	5.50 ± 2.27	4.41 ± 3.17	0.087
Percent of PTV volume enclosed by isodose line (%)	92.09 ± 1.69	91.53 ± 1.77	0.280

### Toxicity outcomes

All 108 patients finished the SBRT treatments. Grade 1–2 acute toxicity reactions occurred in 32 patients, including abdominal pain (3 patients in BED_10_ ≥ 100Gy group, 1 patients in BED_10_ < 100Gy group, *p* = 0.89), fatigue (13 patients in BED_10_ ≥ 100Gy group, 5 patients in BED_10_ < 100Gy group, *p* = 0.53), vomiting and anorexia (22 patients were in BED_10_ ≥ 100Gy group, 6 patients were in BED_10_ < 100Gy group, *p* = 0.92), which could be relieved gradually by symptomatic treatment.

### Liver toxicity

The most serious complication of RILD is the liver failure-resulted death. We reviewed the causes of death of four patients who died within a year after receiving SBRT, among which only one died from liver failure. The patient’ tumor progressed three months after treatment, but his liver function remained normal. The patient only received conservative treatment afterwards. Therefore, we contributed the death of this patient to tumor progression instead of RILD.

In our research, eight patients were diagnosed with RILD through laboratory tests; 4 patients showed classic RILD, and 4 patients showed non-classic RILD (The parameters of RILD patients were shown in Table 6). Among these patients, 1 patient (1/24) was in BED_10_<100Gy group while 7 patients (7/84) were in BED_10_ ≥ 100Gy group (*p* = 0.49). The condition of all patients who were diagnosed with RILD was relieved after symptomatic treatment, and none of these patients died. The analysis of the factors influencing RILD is shown in Table 7. A lower PLT count was associated with an increased risk of RILD on multivariate analysis.

**Table 6:** The parameters of RILD patients before and after SBRT

	ALP		Bilirubin		ALT		AST		ALB		Ascites	
	Pre-	Post-	Pre-	Post-	Pre-	Post-	Pre-	Post-	Pre-	Post-	Pre-	Post-
Classic RILD	131	341	9.8	9.5	28	39	32	32	43	36	–	–
	67	80	12.8	9.9	43	34	43	29	46	35	–	++
	59	327	14.5	8.4	13	18	16	29	44	41	–	+
	113	362	7.8	8.2	16	31	46	38	42	44	–	–
Non-Classic RILD	67	136	22.7	51.6	30	608	28	363	36	40	–	–
	79	81	18.5	35.6	28	17	37	42	39	34	–	–
	79	116	12	16	22	214	27	223	34	33	–	–
	73	147	26.1	51.4	34	749	42	678	36	32	–	+

**Table 7:** Univariate and multivariate logistic regression analysis of RILD

	Univariate logistic regression		Multivariate logistic regression	
Patient details	*p* value	Hazard ratio (95% CI)	*p* value	Hazard ratio (95% CI)
Sex (male/female)	0.116	0.304 (0.069–1.345)		
Age	0.793	0.542 (0.890–1.058)		
Hepatitis type (B/C/alcoholic/others)	0.096	0.019 (0.309–3.269)		
Maximum tumor diameter	0.015	0.096 (0.531–2.092)		
Child-Pugh score (5/6)	0.999	0.000		
ALBI grade	0.398	0.523 (0.116–2.352)		
WBC count	0.009	0.422 (0.220–0.808)		
PLT count	0.006	0.966 (0.942–0.990)	0.034	0.974 (0.950–0.998)
Residual normal liver volume	0.973	1.001 (0.999–1.004)		
Mean dose of whole liver volume minus GTV	0.821	0.997 (0.972–1.024)		
D700	0.002	0.029 (0.901–1.695)		
BED_10_ of PTV	0.696	1.018 (0.930–1.116)		

## Discussion

In this study, we retrospectively reviewed a large group of CP-A HCC patients treated with SBRT over a three-year span as an extended analysis of our previous observations [25]. Compared to prior studies, the OS, PFS, LC and DMFS rates were all satisfactory in this study. Su et al. [4] reported 3- and 5-year OS rates of 73.5 and 64.3% and 3- and 5-year PFS rates of 58.3 and 36.4%, respectively, with doses of 42-46Gy administered in 3–5 fractions and single-fraction doses of 28–30 Gy. Among 77 HCC patients treated with SBRT, Andres et al. [3] found 1- and 2-year OS rates of 81.8 and 56.6%, respectively, and 1- and 2-year LC rates of 99%. The total dose of 45 Gy in 3 fractions was prescribed to the 80% isodose line. Their OS rates were lower than ours, which have been caused by the inclusion of CP-B patients and/or patients with previous treatments. This finding was confirmed by their conclusion that the CP-B classification was associated with a poor prognosis. Moreover, previous treatments may also be an affect factor. Ueno et al. [26] reviewed 296 patients with single nodular HCC ≤ 5 cm with Child-Pugh A between 2001 and 2011 who underwent surgical resection (SR, *n* = 136) and radiofrequency thermal ablation (RFA, *n* = 160), and they found that 5-year OS rates of SR and RFA among all patients were 70.1 and 69.8%, respectively (*P* = 0.14). The 5-year OS was similar to the 5-year cumulative OS in our result.

CP score was an influential factor in OS and PFS in our study, which may be related to the extent to which CP reflects cirrhosis, which is associated with occurrence of complication and tumorigenesis. Moreover, we found that the OS, PFS and DMFS rates also increased significantly with BED_10_ ≥ 100Gy in treating HCC with SBRT, but with no significant difference in the LC rates. The correlation between LC and BEDs in our study was consistent with Ohri’ findings [27], but he didn’t explore the relation between BEDs and survival rates. In addition to initial radiotherapy, the efficacy of subsequent treatment after recurrence was also a factor affecting OS. The relation between PFS and BEDs of our study, we speculate that SBRT could change immune microenvironment, which can be proven by DMFS. This assumption is also supported by some other tumor-related articles. Lee et al. [28] reported that ablative radiotherapy dramatically increased T-cell priming in draining lymphoid tissues, leading to reduction/eradication of the primary tumor or distant metastases. Meanwhile, antitumor immunity significantly contributes to the superior response induced by one dose of 20Gy compared with that induced by 4 doses of 5Gy. Schaue et al. [29] studied the tumor-specific immune response in mice with murine melanoma irradiated with 15Gy administered in different fractionated dose schemes, and the results showed that a single dose of 7.5Gy or higher, but not lower than 5Gy, was immune-stimulatory, as mediated by tumor-reactive T cells. They all showed that under the condition that the total dose remained unchanged, the superior anti-tumor immune response was related with the higher single dose. And it is the same with BEDs, which increase with higher single dose when the total dose remained the same. Therefore, with higher BEDs causing a better anti-tumor immune microenvironment, the recurrence rates of tumor decreased. However, it’s only our hypothesis. In our other studies, we found that in these patients different BED_10_ values lead to different changes of immune system, such as NK cell functions, and further experiments are in progress (data not shown).

The influential factor of RILD is the pretreatment PLT count, as demonstrated by Velec et al. [11]. Some studies shown that the PLT count indirectly reflects the degree of liver cirrhosis by indicating the degree of portal hypertension and hypersplenism [30, 31]. Nozaki et al. [32] performed in vitro and in vivo studies, and they have proven that thrombopoietin promotes liver regeneration and improves liver cirrhosis by increasing the PLT level, indirectly implying that PLT decrease would worsen liver cirrhosis. We think it is the relation between the PLT count and liver cirrhosis, an influential factor of RILD that makes PLT count an influential factor of RILD [33]. In clinical work, we found Child-Pugh score and PLT were not compatible. Degree of liver cirrhosis could affect liver nutrition supply by affecting liver hemodynamics, which may affect liver function. However, liver cirrhosis degree is not the only influence factor of liver function, which may be affected by many other factors, including effective liver volume, hepatic cell function and compensate ability of liver, etc. Moreover, PLT is not a parameter affecting Child-Pugh score. Our result reminds us that besides Child-Pugh score, PLT is also a non-negligible factor.

Though the limited dose of bile duct was not shown in TG-101, previous studies showed that higher dose radiation of bile duct was related with higher risk of stenosis. Barney et al. [34] reported one case of grade 3 biliary stenosis after SBRT where the patient was treated with a dose of 50Gy in five fractions. Takahisa et al. [35] showed that the true threshold for biliary stenosis would be between 40Gy in 5 fractions and 80Gy in 5 fractions, and a special caution was necessary when treating patients with more than 40Gy in 5 fractions. Before this study, we had 2 cases with obstructive jaundice after 50Gy/5f and 54Gy/6f, and their PET-CT showed bile duct stenosis but no active lesion. Since then, we adopted the dose fraction of 49Gy/7f and 48Gy/8f in SBRT of small HCC when the tumor is near to bile duct to reduce this risk. And until now, no patient has had bile duct stenosis by the two doses and fraction sizes above, so we consider they were safe fractionated regimens. Considering both the relation of BEDs and survival rates in our study, we think the trade-off between the efficacy and risk of SBRT in tumors near the bile duct deserves further study, and the optimal dose fractionated regimens needs large-sized samples to explore.

This study has several limitations. First, this was a retrospective study. Second, the number of patients in the two groups is much different, which may cause bias. A relevant prospective clinical trial is in progress (Clinicaltrails NCT 03295500). We hope to get objective and accurate results.

## Conclusion

SBRT is a safe and effective option for CP-A HCC patients, and an increased BED_10_ and lower CP score are associated with improved OS and PFS. The results indicate that a strategy escalating radiation doses over a limited time frame are worth exploring in a prospective clinical trial. Meanwhile, PLT count should be considered, which is a predictive factor of RILD in SBRT of HCC.

## Additional file

Additional file 1: The information of the 108 patients involved in the study. (XLSX 15 kb)

## Data Availability

The information of the 108 patients involved in this study is presented in the Additional file 1. Date sharing is not applicable to this article as no datasets were generated or analyzed during the study.

## Abbreviations

AFP: Alpha fetoprotein
ALP: alkaline phosphatase
BED: Biologically effective dose
CIs: Confidence intervals
CP: Child-Pugh
CR: Complete response
CT: computed tomography
DMFS: distant metastasis-free survival
ECOG: Eastern cooperative oncology group
GTV: Gross target volume
HCC: Hepatocellular carcinoma
HRs: Hazard ratios
LC: Local control
MRI: Magnetic resonance imaging
OS: Overall survival
P: *P*-value
PD: Progressive disease
PET-CT: Positron emission tomography-computed tomography
PR: Partial response
PTV: Planning target volume
RFA: Radiofrequency ablation
RILD: Radiation-induced liver disease
SBRT: Stereotactic body radiation therapy
SD: Stable disease
